# HECT E3s and human disease

**DOI:** 10.1186/1471-2091-8-S1-S6

**Published:** 2007-11-22

**Authors:** Martin Scheffner, Olivier Staub

**Affiliations:** 1 Department of Biology, University of Konstanz, 78457 Konstanz, Germany; 2 Department of Pharmacology and Toxicology, University of Lausanne, Rue du Bugnon 27, 1005 Lausanne, Switzerland

## Abstract

In a simplified view, members of the HECT E3 family have a modular structure consisting of the C-terminal HECT domain, which is catalytically involved in the attachment of ubiquitin to substrate proteins, and N-terminal extensions of variable length and sequence that mediate the substrate specificity of the respective HECT E3. Although the physiologically relevant substrates of most HECT E3s have remained elusive, it is becoming increasingly clear that HECT E3s play an important role in sporadic and hereditary human diseases including cancer, cardiovascular (Liddle's syndrome) and neurological (Angelman syndrome) disorders, and/or in disease-relevant processes including bone homeostasis, immune response and retroviral budding. Thus, molecular approaches to target the activity of distinct HECT E3s, regulators thereof, and/or of HECT E3 substrates could prove valuable in the treatment of the respective diseases.

**Publication history: **Republished from Current BioData's Targeted Proteins database (TPdb; ).

## Preliminary remarks

HECT E3 ubiquitin-protein ligases have been found from yeast to humans and range in size from approximately 80 kDa to more than 500 kDa. They are characterized by the HECT (*h*omologous to *E*6-AP *C-t*erminus) domain, a C-terminal region of approximately 350 amino acids in length with significant similarity to the C-terminus of E6-AP [1,2]. The HECT domain mediates the interaction with cognate E2 ubiquitin conjugating enzymes and, via an evolutionally conserved cysteine residue, forms thioester complexes with ubiquitin [1-6]. Since the ability to form ubiquitin thioester complexes in the presence of E2s is required for their E3 ligase activity, it is commonly assumed that, unlike members of other E3 families (i.e. RING finger E3s, U box E3s), HECT E3s play a direct catalytic role in the final attachment of ubiquitin to substrate proteins [1-9].

**Figure 1:**
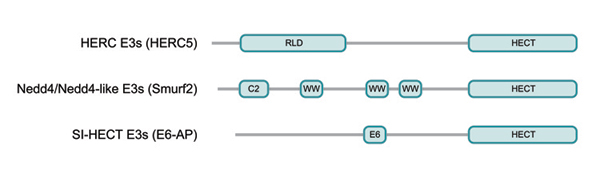
The family of HECT E3s. All members of the HECT E3 family are characterized by the C-terminal HECT domain, which consists of approximately 350 amino acid residues and represents the catalytic domain. The HERC family comprises six members, which are characterized by the presence of one or several RLD domains (as representative, the structure of HERC5 is schematically shown). The Nedd4/Nedd4-like family has nine members that are characterized by an N-terminal C2 domain and the presence of several WW domains (as representative, the schematic structure of Smurf2 is shown). The schematic structure of E6-AP, the founding member of the HECT E3 family, is shown as representative of the third subfamily (“E6” denotes the binding site of E6-AP for the HPV E6 oncoprotein). Members of this subfamily (SI-HECT E3s) are characterized by the notion that they contain neither RLDs nor WW domains.

While the HECT domain represents the catalytic domain of HECT E3s [1-3], the substrate specificity of these proteins is assumed to be determined by their respective N-terminal extensions (see figure 1). Based on the presence of distinct amino acid sequence motifs within these N-terminal extensions, human HECT E3s can be grouped into three subfamilies: HECT E3s with RLDs (*R*CC1-*l*ike *d*omains) (which are termed HERC (*HE*CT and *RC*C1-like domain) E3s) [10], HECT E3s with WW domains (termed Nedd4/Nedd4-like E3s) [11,12] and HECT E3s that neither contain RLDs nor WW domains (termed SI(ngle)-HECT E3s) (see figure 1). Since RLDs [13-16] and WW domains [17-19] represent known protein–protein interaction domains, their presence provides some information about potential interaction partners of the respective E3.

Database analyses indicate that the human genome encodes 28 different HECT proteins (K. Hoffmann and H. Scheel, personal communication). Since all of these proteins contain the catalytic site cysteine residue, it can be assumed that all of these have E3 activity. However, although this will not be further addressed, one should keep in mind that the sheer size of HECT proteins could indicate that, at least in some cases, the function of HECT proteins may not be restricted to ubiquitylation (i.e. HECT E3s are multifunctional proteins). In the following, we will focus on those HECT E3s that have been associated with human disease, disease-relevant processes, and/or abnormal phenotypes in mice, and will discuss these within the context of the abovementioned HECT subfamilies. For a summary of the HECT E3s discussed in this review see Table 1.

**Table 1:** Summary of HECT E3s discussed

	**Chromosomal localization**	**Potential substrate proteins**	**Mouse model**	**Human disease**
**HERC1**	15q22	TSC2	n.a.	Tuberous sclerosis complex (?)
**HERC2**	15q13	Unknown	Neuromuscular and spermatogenic defects; juvenile lethality	?
**HERC5**	4q22	Unknown	n.a.	?
**Nedd4-1**	15q21	PTEN, ENaC, viral Gag proteins, numerous membrane transport proteins or receptors	n.a.	Liddle's syndrome (?) Retroviral infection (?) Cancer (?)
**Nedd4-2**	18q21	ENaC, Nav 1.5, numerous membrane transport proteins	n.a.	Liddle's syndrome (?) Retroviral infection (?)
**Smurf1**	7q21-31	Smad1, Smad5	Bone homeostasis	Bone homeostasis (?)
**Smurf2**	17q22-23	Smad1, Smad2, SnoN, TGF-β receptor	Double knockout with Smurf1; embryonic lethal	Cancer (?)
**Itch**	20q11.22-11.23	JunB, c-jun, PKC-θ PLC-γ1, p63, p73	Defects in Th2 cell differentiation and tolerance	Th2 cell allergy (?)
**E6-AP**	15q11-13	Bak, Blk, HHR23, Mcm7, AIB1 In complex with the HPV E6 oncoprotein: p53, PDZ domain-containing proteins (e.g. hDlg, Scribble, MAGI-1), NFX1-91	AS-like phenotype	AS Cervical cancer
**EDD**	8q22.3	TopBP1, Paip2	Defects in yolk sac and allantoic vascular development; embryonic lethal	Cancer (?)
**HECTH9**	Xp11.22	histones, Mcl-1, c-Myc, p53	n.a.	Cancer (?)

n.a., not available; ?, link to human disease has not been reported; (?), the respective HECT E3 has been linked to the respective disease but it remains unclear as to whether it is causally involved. For references, see text.

## Localization and function

### HERC E3s

The human HERC E3 subfamily has six members that, based on molecular mass, can be further divided into “large” HERCs (with a molecular mass of more than 500 kDa, i.e. HERC1 and HERC2) and “small” HERCs (with a molecular mass of approximately 100–120 kDa). The mRNAs for the various HERCs are expressed in many tissues and the HERC proteins appear to be mainly localized to the cytoplasm and, from there, to membranous or vesicular structures. This suggests that, in general, HERC E3s are involved in membrane traffic pathways (for a recent review on HERC E3s, see [10]).

The RLD of HERC E3s consists of usually seven repeats of 50–70 amino acids in length and was first described for the mitotic regulator RCC1 [13]. Structural studies revealed that RCC1 adopts a seven-bladed β-propeller structure with each repeat corresponding to one blade [14]. The RCC1 propeller structure serves at least two functions: one side of the propeller binds to the GTP-binding protein Ran and acts as a guanine nucleotide exchange factor, while the opposite side interacts with core histones, thus mediating the interaction of RCC1 with chromatin [15,16]. Although it remains to be shown if the RLDs of HERC E3s also serve dual functions, recombinant HERC1 expressed in insect cells was shown to bind to GTP-binding proteins of the ARF and Rab family [20]. However, it remains unclear if these interactions are related to the E3 activity of HERC1, since ARF/Rab do not appear to be ubiquitylated by HERC1. Recently, HERC1 was reported to interact with TSC2, a GTPase-activating protein of the Rheb GTPase, in murine and human cells, and to target it for degradation [21]. TSC2 negatively affects the mTOR pathway and has been associated with tuberous sclerosis complex, a hereditary disease characterized by hamartoma formation in various organs. It remains to be shown, however, whether HERC1 is involved in the development of this disease.

Although substrates or interacting partners of HERC2 have not been described, the protein has nevertheless received considerable attention. Firstly, mutations in the *Herc2* gene have been linked to pathophysiological phenotypes in mice (see section on *Disease, mutation, expression, knockout* - *HERC E3s*) [22,23]. Secondly, the human *HERC2* gene is located on chromosome 15q11-13, which is known as the Prader-Willi/Angelman region [24]. This region comprises approximately four megabases, is bounded by duplicons of the *HERC2* gene that may predispose to chromosomal rearrangements and contains a bipartite imprinting center (and, thus, maternally and paternally imprinted genes). Prader-Willi syndrome (PWS) and Angelman syndrome (AS) represent two clinically distinct neurodevelopmental disorders, with PWS resulting from paternal genetic deficiency and AS from maternal genetic deficiency [25]. However, since the *HERC2* gene is not imprinted [25], loss or alteration of *HERC2* expression and, thus, of HERC2 function do not appear to be involved in the development of PWS and AS, respectively.

With the exception of HERC5, very little is known about the potential physiological functions of “small HERCs” [10]. HERC5 was originally isolated in a yeast two-hybrid screen as a Cyclin E binding protein, though the physiological relevance of this observation remains unclear [26]. More recently, it was shown that *HERC5* expression is upregulated in response to pro-inflammatory stimuli and that HERC5 acts as an E3 ligase for ISG15, a ubiquitin-like protein that is expressed upon stimulation of cells with interferon [10,27,28]. These data indicate that HERC5 plays an important role in the immune response.

### Nedd4/Nedd4-like E3s

The human Nedd4/Nedd4-like E3 subfamily consists of nine members with respective orthologs in mice. Family members share a common structure: an N-terminal calcium-dependent phospholipid binding C2 domain, two to four WW domains (a highly conserved protein domain that binds to proline-rich regions) and the HECT domain (see figure 1) (for recent reviews on Nedd4/Nedd4-like proteins see [11,12]). The diversity of this family is further enhanced by alternative splicing of some (possibly all) family members. Nedd4/Nedd4-like E3s are involved in various pathways including endocytosis [29], degradation of membrane proteins [30], control of cell growth [31] and virus budding [32]. It is, therefore, not surprising that Nedd4/Nedd4-like E3s have been involved in several pathologies including hypertension [30], cancer [33] and defects in the immune system [34]. In the following, we will focus on Nedd4-1, Nedd4-2, Smurfs and Itch. All these appear to be ubiquitously expressed, though within the tissues they are likely to be differentially expressed, as shown for Nedd4-1 and Nedd4-2 [35-38]. They are mostly cytosolic proteins [39,40], although Smurf2, for example, can be localized into the nucleus when transiently expressed [41]. In addition, these proteins can bind via their C2 domain to cell membranes, as shown for Nedd4-1, which can interact with annexin 13 in a Ca^2+^-dependent fashion and thereby be targeted to the apical membrane of epithelial MDCK cells [42].

Nedd4-1, also referred to as Nedd4 (*n*euronal precursor cell *e*xpressed *d*evelopmentally *d*ownregulated 4), is the founding member of the Nedd4/Nedd4-like E3 family and was originally identified in a subtractive screen using mRNAs derived from different stages of brain development [43]. Although Nedd4-1 was originally described as a protein containing three WW domains [44], it is now evident that there exist multiple alternative transcripts encoding different forms of Nedd4-1 that do or do not have the C2 domain and which possess varying numbers of WW domains.

Both Nedd4-1 and Nedd4-2 (a close relative) have been proposed to play a role in Liddle's syndrome (a rare hereditary form of hypertension), the budding of retroviruses including HIV-1 and HTLV-1 (which are etiologically associated with AIDS and adult T-cell leukemia, respectively) and T-cell receptor (TCR) signaling [45-47]. Known ubiquitylation substrates of Nedd4-1 or Nedd4-2 include ENaC (Liddle's syndrome), viral GAG proteins (retroviral budding), PKC-θ and PLC-γ1 (TCR signaling), and PTEN [31,45-47]. In many cases, PY motifs in the substrate proteins serve as binding sites for the WW domains of Nedd4-1 and/or Nedd4-2.

**Figure 2:**
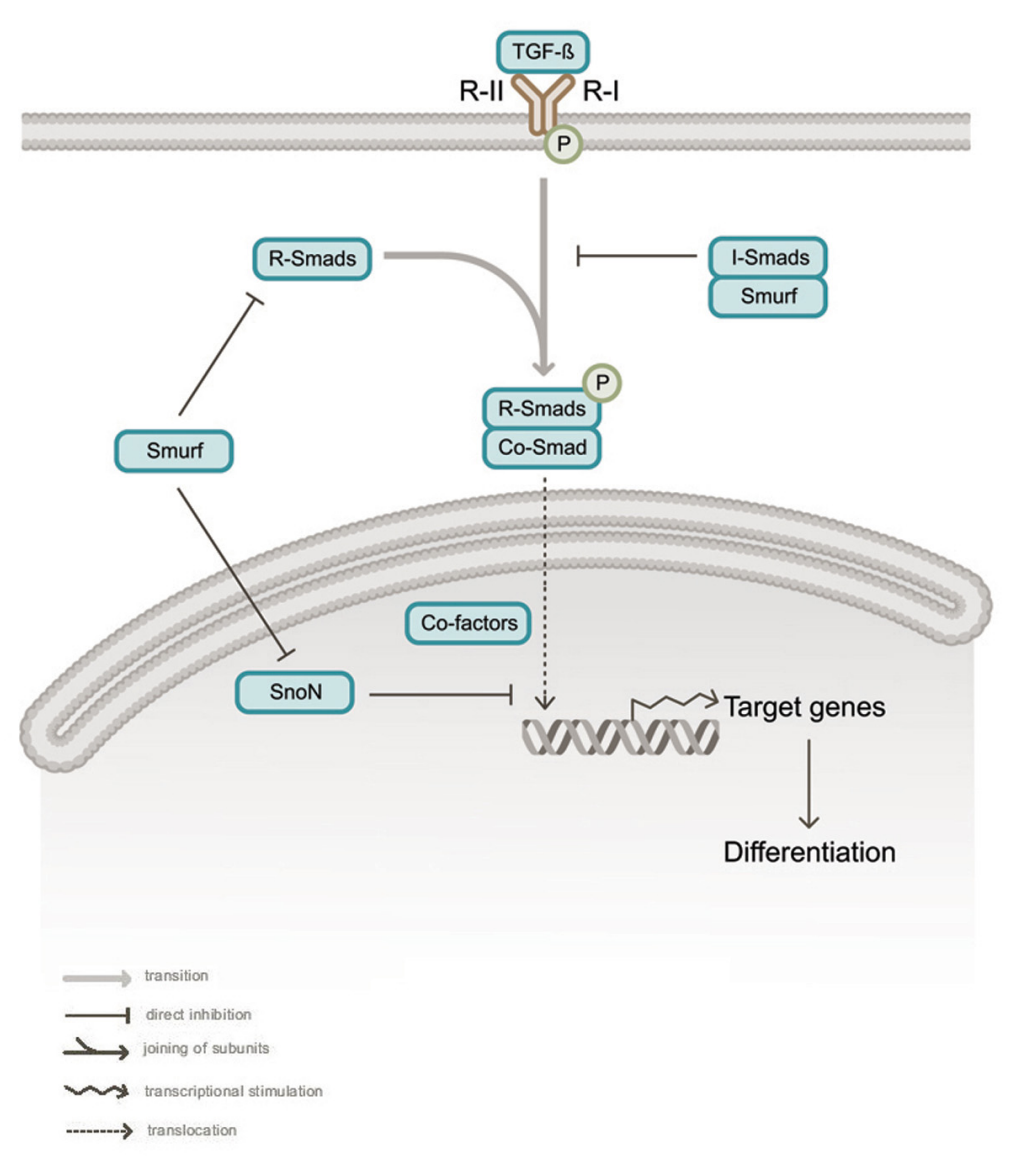
Role of Smurfs in the TGF-β/BMP pathways. TGF-β ligand stimulates heterodimerization of type I and type II Ser/Thr kinase receptors (labeled R-I and R-II), leading to phosphorylation of type I receptor by type II receptor. This recruits receptor regulated Smads (R-Smads), which become phosphorylated. Upon phosphorylation, R-Smads interact with the common Smad (co-Smad), Smad4, and the complex translocates into the nucleus, where it interacts with co-factors and stimulates transcription of genes involved in differentiation. The pathway is negatively regulated by inhibitory Smads (I-Smads), by SnoN and by Smurfs. Smurfs can interact with and ubiquitylate R-Smads and can be recruited by I-Smads to the receptor, where they induce receptor ubiquitylation and internalization. Furthermore, Smurfs are also involved in SnoN ubiquitylation, and thus are also able to act as positive regulators of this pathway.

Smurfs (Smurf1 and Smurf2) were originally identified in a yeast two-hybrid screen as proteins that, via WW domains, interact with a PY motif in Smad-1, a protein involved in TGF-β/BMP signaling [48]. The TGF-β superfamily comprises approximately 40 members including TGF-β, activins, nodals and the bone morphogenetic proteins (BMPs) [49], and mutations in TGF-β pathway components are associated with several human diseases including cancer and osteoporosis. TGF-β ligand receptors are heterodimers composed of type I and type II class receptors and have Ser/Thr kinase activity. Heterodimerization is induced by ligand binding and results in type II receptor-mediated phosphorylation of the type I receptor, which in turn becomes activated and phosphorylates serine residues on receptor-regulated Smads (R-Smads) (Smad-1, -2, -3, -5, and -8) (see figure 2). Smad-1, -5 and -8 are involved in the BMP pathway, whereas Smad-2 and Smad-3 are signaling components of the TGF-β pathway. Once phosphorylated, R-Smads complex with the common co-Smad, Smad-4, and translocate into the nucleus, where they regulate transcription. Furthermore, inhibitory Smads (I-Smads) (Smad-6 and Smad-7) interfere with TGF-β and BMP signaling by competing with R-Smads for association with type I receptors or by targeting receptors for ubiquitin-mediated degradation (see figure 2).

Smurf1 and Smurf2 act at different levels of the TGF-β/BMP signaling pathways. Smurf1 targets the BMP signaling proteins Smad-1 and Smad-5 for ubiquitin-dependent degradation [48], whereas Smurf2 associates with Smad-1 and Smad-2, inducing a decrease in the levels of these two proteins [50]. Importantly, Smad-2 activation results in an increased interaction with Smurf2, leading to its proteasome-mediated degradation in the nucleus [51], suggesting that Smurf2 is important for termination of TGF-β signaling. However, Smurf2 may also facilitate TGF-β signaling, since Smad-2 recruits Smurf2 to the transcriptional co-repressor SnoN [52], an inhibitor of the TGF-β transcriptional response, resulting in SnoN ubiquitylation and degradation (see figure 2). Yet another mechanism involves the I-Smad Smad-7. Upon binding, Smad-7–Smurf2 are exported from the nucleus and interact with the TGF-β receptor. In concert with the E2 enzyme UbcH7, this results in Smad-7 ubiquitylation, triggering internalization and degradation of both Smad-7 and the receptor [41]. Similar to Smurf2, Smurf1 is also recruited by Smad-7 to the TGF-β receptor [53]. Finally, both Smad-6 and Smad-7 can mediate translocation of Smurf1 to BMP receptors, leading to ubiquitylation and degradation of these receptors [54].

Itch (also referred to as AIP4) was originally identified as the protein encoded by the *itchy* locus (located on chromosome 2) in non-agouti lethal 18H mice [55]. The phenotype of both these and genetically engineered Itch null mice indicate that Itch is crucially involved in Th2 cell differentiation and anergy. In response to stimulation with anti-CD3, or anti-CD3 plus anti-CD28, T-cells can undergo chronic activation, which is accompanied by increased production of IL-4 and IL-5, resulting in a biased differentiation of CD4^+^ cells into Th2 cells [34]. During T-cell differentiation, Itch binds via its WW domains to the PPXY motif of Jun-B and/or c-Jun, promoting their ubiquitylation and subsequent proteasomal degradation. Since the transcription factors Jun-B and c-Jun are intrinsically involved in the regulation of Th2 cytokine expression (including that of IL-4), this indicates that Itch is involved in the negative control of Th2 cell differentiation [34]. Indeed, the Th2-dependent serum concentrations of IgG1 and IgE are increased in itchy mice [34].

T-cell anergy, a process contributing to self-immune tolerance, is a state of unresponsiveness that is achieved when the TCR is engaged without co-stimulation of accessory molecules such as the CD86 receptor [56,57]. In the absence of co-factors, TCR engagement is sufficient to stimulate mobilization of intracellular free calcium ions, leading to activation of calcineurin. In turn, calcineurin dephosphorylates the transcription factor NF-AT1, resulting in its activation and subsequent stimulation of the transcription of “anergy genes”, whose products keep T-cells in an anergic state [58]. Among several signaling proteins and other ubiquitin-protein ligases (Grail, Cbl-B), Itch and Nedd4 are induced by this calcium/calcineurin signaling pathway [47]. Itch and Nedd4 become membrane-associated (most likely via their C2 domain) and bind and monoubiquitylate PKC-θ and PLC-γ1, both of which are important for TCR signaling and immunological synapse formation. Monoubiquitylated PKC-θ and PLC-γ1 are in turn targeted via the ESCRT pathway into lysosomes and degraded [47].

The E3 activity of Itch is regulated by phosphorylation. Fyn kinase-mediated tyrosine phosphorylation has a negative effect on Itch activity [59], whereas phosphorylation of Itch by the MEKK1/JNK1 pathway results in Itch activation and, consequently, in increased turnover of Jun-B and c-Jun (60). Furthermore, results obtained with mice expressing MEKK1^ΔKD^, a catalytically inactive MEKK1 mutant, are consistent with the notion that phosphorylation of Itch is required to negatively control Th2 cell activation. Additionally, Th2 cells that either express an inactive MEKK1 mutant or are devoid of JNK1 or Itch cannot become immune tolerized, whereas Th1 tolerance is not affected [61]. Thus, the MEKK1/JNK1/Itch pathway is important for Th2 cell differentiation and Th2 tolerance (T-cell anergy).

Itch has also been implicated in TNFα signaling [62]. The biological outcome of TNFα signaling depends on the balance between the NF-κB and JNK signaling pathways, with NF-κB promoting survival and JNK enhancing cell death. It has been shown that TNFα-mediated JNK activation results in phosphorylation and activation of Itch. Itch then targets the NFκB-induced anti-apoptotic protein c-FLIP for ubiquitin-dependent degradation. Importantly, JNK1 or Itch null mice are resistant to TNFα-induced acute liver failure and cells derived from these mice are not proficient in inducible c-FLIP degradation [62].

Itch has also been shown to bind to, ubiquitylate and thereby negatively regulate p63 and p73, which are members of the p53 family of transcription factors, suggesting a role for Itch in the control of the cell cycle and apoptosis [63-65]. Interestingly, p73 was also shown to be ubiquitylated by Nedd4-2 [66]. Furthermore, p53 has recently been shown to be ubiquitylated by WWP1, another Nedd4/Nedd4-like E3 enzyme [67].

### SI-HECT E3s

The best studied members of this HECT E3 subgroup are E6-AP, EDD and HECTH9, all of which have been associated with human disease. mRNA of E6-AP, which represents the founding member of the HECT family of E3s [1,2,68], is ubiquitously expressed and the protein is found in the cytoplasm and nucleus. Mutations within the encoding gene, *UBE3A* (located on chromosome 15q11-13), that interfere with the E3 activity of E6-AP have been etiologically associated with the development of AS [69,70]. Importantly in this respect, imprinting of the *UBE3A* gene is restricted to the brain, whereas it is biallelically expressed in all other tissues [71,72]. E6-AP (*E6*-*a*ssociated *p*rotein) was originally isolated as a protein that binds to the E6 oncoprotein of human papillomaviruses (HPVs) etiologically associated with malignant cancers of the anogenital tract, most notably cervical cancer [68,73].

E6-AP is hijacked by E6 to target several cellular proteins for ubiquitin-dependent degradation. Known substrates of the E6–E6-AP complex include the tumor suppressor p53, the PDZ domain-containing protein Scribble and NFX1-91, a transcriptional repressor of the gene encoding hTERT, the rate-limiting catalytic subunit of telomerase [74-76] (for reviews, see [77,78]). Importantly, cell culture studies and studies with transgenic mice clearly indicate that the ability of E6 to interact with both E6-AP and PDZ domain-containing proteins is crucial for its oncogenic potential (see *Disease, mutation, expression, knockout* - *SI-HECT E3s*). Furthermore, E6-AP may not only be utilized by E6 but, in addition, may represent a direct target for E6, since binding of E6 targets E6-AP for ubiquitylation and degradation [79]. However, the physiological relevance of this observation remains unclear. Similarly, since cell culture systems that allow the efficient propagation of HPVs are not available, the significance of the E6–E6-AP interaction for the viral life cycle is not known.

In contrast to the situation in HPV-positive cancer cells, the physiological role of E6-AP in normal (i.e. HPV-negative) cells remains elusive. Several E6-independent substrates of E6-AP have been reported, including HHR23A and HHR23B (the human orthologs of *Saccharomyces cerevisae* Rad23), Blk (a member of the Src family of non-receptor tyrosine kinases), Bak (a human pro-apoptotic protein), Mcm7 (which is involved in DNA replication) [77,78] and AIB1 (a steroid receptor coactivator) [80]. However, the physiological relevance of these interactions remains to be shown.

Human EDD (*E*3 identified by *d*ifferential *d*isplay) represents an ortholog of HYD, the *Drosophila* hyperplastic discs tumor suppressor gene product. EDD mRNA is expressed in many tissues and is frequently overexpressed in breast and ovarian cancers, suggesting a possible role in tumor development [81-83]. The EDD protein consists of 2799 amino acid residues and contains three putative nuclear localization sequences, an N-terminal UBA domain (which mediates EDD's interaction with ubiquitin), a central UBR1-like zinc finger motif and a PABC domain (a peptide binding domain found in the poly(A)-binding protein PABP) [81,84-87]. EDD has been shown to bind to and ubiquitylate the topoisomerase II beta-binding protein TopBP1, and to bind to and potentiate progesterone receptor-mediated transcriptional transactivation [85,86]. More recently, it has been reported that EDD binds to the DNA damage checkpoint kinase Chk2 and acts upstream of Chk2 [88], supporting the notion that EDD is involved in pathways regulating the DNA damage response. Furthermore, it has been demonstrated that downregulation of PABP results in EDD-mediated degradation of the PABP-associated protein Paip2, which interferes with translation by displacing PABP from mRNA [89]. This indicates that EDD is involved in controlling the activity of PABP and, thus, in controlling translational efficiency.

HECTH9 mRNA is expressed in many tissues and the protein appears to be mainly located in the nucleus. Importantly, HECTH9 is overexpressed in several different tumors, suggesting that deregulation of HECTH9 activity contributes to cancerogenesis [90]. HECTH9 was initially reported to interfere with p53-mediated transcriptional transactivation [91]. However, the physiological significance of this observation remained unclear, since it turned out that the protein studied (termed UREB1 at the time) represented a significantly N-terminally truncated version of HECTH9. Recently, several groups reported on the identification of substrate proteins of full-length HECTH9. These included p53, histones, the anti-apoptotic protein Mcl-1 and the proto-oncoprotein c-Myc (note that the respective authors referred to HECTH9 with different names, including E3 histone, ARF-BP1 (ARF-binding protein 1) and MULE (Mcl-1 ubiquitin ligase E3)) [90,92-94]. Note that p53, Mcl-1 and c-Myc were identified as substrates *in vivo* using ubiquitylation assays in overexpression experiments in cultured cells, and that these findings were supported by *in vitro* assays. HECTH9 has only been shown to ubiquitylate histones using *in vitro* ubiquitylation assays.

HECTH9 consists of 4374 amino acid residues and contains three domains known to serve as protein–protein interaction sites: a BH3 domain, a WWE domain and a UBA domain (for a recent review on HECTH9, see [95]). The BH3 domain is required for the interaction of HECTH9 with Mcl-1 [94], whereas the interactions sites for p53 and c-Myc have not been mapped in detail. Interestingly, while HECTH9-mediated ubiquitylation targets p53 and Mcl-1 for proteasomal degradation, c-Myc is not targeted for degradation by HECTH9. Instead, HECTH9 modifies c-Myc with ubiquitin chains that are linked via lysine residue 63 (K63) of ubiquitin [90] and serve non-proteolytic roles [7-9]. Specifically, HECTH9-mediated ubiquitylation of c-Myc appears to be required for transactivation of multiple target genes of c-Myc and induction of cell proliferation. Taken together, the available data indicate that HECTH9 has pro-proliferative activities.

## Disease, mutation, expression, knockout

### HERC E3s

To date, none of the members of the HERC E3 subfamily has been etiologically associated with human disease and transgenic HERC mice have not been reported. In the late 1990s, however, it was shown that so-called *rjs* (*r*unty, *j*erky, *s*terile) mice harbor mutations in the *Herc2* gene [23,96]. *Rjs* mice (commercially available from The Jackson Laboratory, Maine, USA) have neuromuscular and spermatogenic abnormalities and are characterized by defects in growth, movement coordination (jerky gait) and fertility. Importantly, one of the *rjs* mutants isolated expresses a C-terminally truncated form of HERC2 lacking part of the C-terminal HECT domain [96]. This indicates that loss of HERC2 E3 activity is responsible for the observed phenotype. Although deregulated expression of human HERC2 has not been causally associated with any human disease, identification of HERC2 substrate proteins may eventually reveal whether or not HERC2 plays a role in human disease.

### Nedd4/Nedd4-like E3s

Liddle's syndrome is characterized by early onset of severe hypertension, hypokalemia, metabolic alkalosis and low circulating levels of renin and aldosterone [97,98]. Patients are treated with a low Na^+^ diet and administration of triamterenes, which are specific inhibitors of ENaC. All the available data suggest that Na^+^ reabsorption by epithelia of the distal nephron is deregulated in this disease. Normally, such cells express at their apical side (facing the urinary compartment) ENaC, which allows entry of Na^+^ into the cell, and on the basolateral side the Na^+^,K^+^-ATPase, which extrudes Na^+^ out of the cell into the blood compartment. This Na^+^ transport is highly regulated by the renin-angiotensin-aldosterone system. In Liddle's syndrome patients, genes encoding ENaC subunits are mutated [99,100]. ENaC is a transmembrane protein that is composed of three homologous subunits, which assemble into tetramers (2α1β1γ). The C-terminus of each subunit contains PY motifs that serve as binding sites for the WW domains of Nedd4-1 and/or Nedd4-2 [101]. Upon binding, Nedd4-1/Nedd4-2 ubiquitylate the N-termini of the ENaC subunits, leading to ENaC internalization and degradation by the endosomal/lysosomal system [36,37,45]. In Liddle's syndrome patients, a PY motif is mutated or deleted either in the β- or γ-subunit [99,100], leading to impaired interaction with Nedd4-1/Nedd4-2 and, consequently, accumulation of ENaC at the plasma membrane.

The majority of evidence, including that derived from co-localization [38] and RNAi studies [102], indicates that Nedd4-2 is the physiologically relevant regulator of ENaC in the kidney. Importantly, Nedd4-2 is a substrate of the aldosterone-induced kinase Sgk1, a key regulator of Na^+^ transport. Phosphorylation of Nedd4-2 creates binding sites for 14-3-3 proteins, which interfere with ENaC–Nedd4-2 interaction, causing an increase in channels at the cell surface [103-105]. Furthermore, polymorphisms in the Nedd4-2 gene have been associated with hypertension [106]. However, definite proof that Nedd4-2 controls Na^+^ transport in the distal nephron will have to await its inactivation and analysis in a conditional transgenic mouse knockout model or unambiguous genetic linkage of the Nedd4-2 locus to Liddle's syndrome or other forms of hypertension.

Retroviral budding takes advantage of the cellular machinery that targets proteins for lysosomal degradation via the multiple vesicle body (MVB) pathway (for reviews, see [107,108]). Essentially, late domains of viral GAG proteins contain a P(S/T)AP motif, a PY motif, or a tyrosine-based sorting motif (YP_x_(n)L), which recruit components of the MVB machinery (e.g. Tsg101) and promote virus budding. Although viral PY motifs bind to Nedd4 family members, it is not yet clear how this promotes particle release. Ubiquitylation of GAG could recruit the MVB machinery, for example via binding to Tsg101 or to other ubiquitin binding proteins of the ESCRT pathway. Alternatively, since Nedd4 and Tsg101 can interact with each other, Nedd4 could recruit Tsg101 to the GAG protein, inducing virus budding [109]. Furthermore, studies using HTLV-1 GAG, which contains both a PY motif and a P(S/T)AP motif (which binds Tsg101), showed that mutation of the PY motif leads to accumulation of the virus at the plasma membrane, whereas mutation of the P(S/T)AP motif leads to accumulation in endosomes. This suggests that HTLV-1 first interacts at the plasma membrane with Nedd4 and later with Tsg101 in endosomes [32]. In any case, Nedd4 family members appear to play a crucial role in retroviral budding and, thus, in the spreading of these viruses.

TGF-β has both tumor suppressive and tumor promoting effects [110]. Thus, both inappropriate inactivation and activation of Smurfs could contribute to cellular transformation and cancerogenesis. Indeed, Smurf2 overexpression in esophageal squamous cell carcinoma correlates with poor prognosis [33]. Similarly, oncogenic missense mutations in the *Smad-2* or *Smad-4* genes target the respective mutant proteins for increased ubiquitylation and degradation when compared with the wild-type proteins, thereby interfering with TGF-β signaling [111]. Although the E3s involved in the degradation of the Smad mutants have not yet been identified, it was shown that RNF11, a RING finger E3 that is highly expressed in invasive breast cancer [112], interacts with Smurf2 and targets it for ubiquitylation and degradation. Furthermore, RNF11 can interfere with Smurf2-mediated ubiquitylation of the TGF-β receptor. By blocking Smurf2 activity, RNF11 could enhance TGF-β signaling and its tumor-promoting activities in certain tissues.

Since both TGF-β and BMPs signal through Smads, it is not surprising that Smurfs, especially Smurf1, affect bone homeostasis. Overexpression of Smurf1 interferes with BMP-induced osteoblast differentiation [113,114], whereas RNAi-mediated downregulation of Smurf1 expression, or expression of catalytically inactive Smurf1, enhances osteoblast differentiation [113,115]. Furthermore, transgenic mice overexpressing Smurf1 (generated by the Chen Group, University of Rochester, USA) display significantly reduced bone formation [114]. These findings are in agreement with the notion that Smurf1 controls the levels of Smad-1 and/or Smad-5 (see *Localization and Function - Nedd4/Nedd4-like E3s*), as well as the levels of the osteoblast-specific transcription factor Runx2 [114-116]. The strongest evidence supporting a role for Smurfs in bone homeostasis comes from Smurf1 null mice (generated by the Zhang Group, National Cancer Institute, Bethesda, USA) [117]. These mice are viable, develop normally and have a similar life expectancy to wild-type mice. However, starting from approximately four months of age, Smurf1 null mice show an increase in bone mass caused by enhanced osteoblast activity, indicating that Smurf1 is important for bone-forming activities in mature osteoblasts. Surprisingly, neither BMP signaling nor Runx2 activity seem to be affected in Smurf1 null mice; rather the JNK signaling pathway is constitutively active, resulting in enhanced expression of JNK-responsive genes. Smurf1 interacts with MEKK2, an upstream activator of JNK, and targets it for ubiquitin-dependent degradation, suggesting that deregulated MEKK2 activity is responsible for the phenotype of Smurf1 null mice. Indeed, expression of constitutively active MEKK2 or JNK or inactive MEKK2 in osteoblasts demonstrates that these kinases regulate osteoblast activity. The lack of effect of Smurf1 inactivation on the BMP pathway could be explained by compensatory functions of Smurf2. Indeed, Smurf1 null mice show increased Smurf2 expression. Furthermore, Smurf1/Smurf2 double knockout mice (generated by the Zhang Group, National Cancer Institute, Bethesda, USA) are embryonic lethal, supporting the idea that Smurf1 and Smurf2 have overlapping or compensatory functions [117]. Although involvement of Smurf1 in human pathologies with dysregulated bone homeostasis (such as osteoporosis) remains to be demonstrated, the fact that Smurf1 inactivation has no effect on the maintenance of skeletal integrity could aid development of therapeutic strategies for treating age-related bone loss such as osteoporosis.

In *Itchy* mice (generated by the Copeland/Jenkins Group at the National Cancer Institute, Frederick, USA; see *Localization and Function* - *Nedd4/Nedd4-like E3s*), Itch is inactivated by gene inversion, alongside promoter inactivation of the agouti gene, leading to a darker color coat [55]. Depending on the genetic background, the mice present two different but related phenotypes: on a JC/Ct background they display an inflammatory disease of the large intestine, whereas on a C57L/6J background they present a fatal disease involving changes in lung, spleen, lymph nodes, skin, ear, thymus and stomach. In each organ, the phenotype suggests hyperactivation of processes typical for chronic inflammation. Moreover, *Itchy* mice are characterized by skin and ear scarring, caused by constant itching when older than 16 weeks, and display larger spleens and lymph nodes, likely due to hyperproliferation of lymphocytes. This phenotype points to a crucial role for Itch in the negative control of the immune system. Indeed, as discussed in *Localization and Function - Nedd4/Nedd4-like E3s*, the Itch pathway appears to be particularly important for the regulation of Th2 cell differentiation; thus, Th2 tolerance and defects in this pathway could contribute to Th2 cell-mediated allergies. These notions are supported by experiments performed with T-cells derived from *Itchy* mice [34].

### SI-HECT E3s

E6-AP represents a prime example supporting the notion that deregulation of components of the ubiquitin conjugation system contributes to human disease: inappropriate activation of E6-AP ("gain of function") contributes to the development of cervical cancer and inactivation of E6-AP ("loss of function") results in AS.

Infection with certain HPV types represents the most significant risk factor for the development of cervical cancer, the second most frequent cancer in women worldwide with approximately 400,000 new cases each year (for review see [73]). The suggested ability of the HPV E6 oncoprotein to utilize E6-AP to target p53 and other cellular proteins for degradation is supported by several observations. Firstly, transgenic mouse models indicate that HPV-induced tumorigenesis is crucially dependent on both ablation of p53 activity and the interaction of E6 with PDZ domain proteins [118,119]. Secondly, in contrast to many other tumor types (approximately 40% of all human tumors harbor a mutated p53 gene), the p53 gene is very rarely mutated in cervical carcinomas [73,78]. Thus, E6/E6-AP-induced degradation of p53 can be considered functionally equivalent to inactivation of p53 by mutation of the p53 gene, although the situation in HPV-positive cancers may be somewhat more complicated (for detailed discussion of this issue, see [78]). Thirdly, interference with E6-AP expression by antisense RNA-based approaches or by RNAi results in accumulation of p53 and activation of its transcriptional and growth-suppressive properties [120-123].

AS was first described in 1965 by the pediatrician Harry Angelman [124,125]. It is a genetic disorder with an incidence of approximately 1 in 10000 to 1 in 40000 and is characterized by mental retardation, movement or balance disorder, characteristic abnormal behaviors and severe limitations in speech and language. As discussed in *Localization and Function - SI-HECT E3s*, AS has been linked to chromosome 15q11-13, which contains the *UBE3A* gene [25,69,70]. All of the genetic abnormalities associated with AS affect expression of the maternal *UBE3A* gene and/or the ubiquitin ligase activity of E6-AP. Development of AS appears to be the result of several genetic mechanisms, with deletion of the 15q11-13 region of the maternal chromosome accounting for approximately 70 percent of cases. Other mechanisms include uniparental paternal disomy, defects in imprinting and single point mutations in the *UBE3A* gene [25,125,126]. In this context, it should be noted that E6-AP affects the activity of nuclear hormone receptors and that this property does not appear to be related to its E3 function [127]. However, the relevance of this function for the development of AS or cervical cancer is unclear (e.g. this property is not affected in E6-AP mutants derived from AS patients with point mutations in the *UBE3A* gene). The notion that loss of E6-AP activity is responsible for the development of AS is strongly supported by transgenic mouse models [128,129]. Furthermore, studies in mice have shown that, similar to the situation in humans, the murine *Ube3a* gene encoding E6-AP is biallelically expressed in all somatic cells with the exception of Purkinje cells (cerebellum), hippocampal neurons and mitral cells of the olfactory bulb, in which the paternally derived *Ube3a* gene is silenced [130]. Finally, it should be noted that in E6-AP null mice (generated by the Beaudet Group, Baylor College of Medicine, USA and commercially available from The Jackson Laboratory, Maine, USA and the Wagstaff Group, University of Virginia, USA), cytoplasmic levels of p53 are significantly increased in postmitotic neurons. However, since all available data indicate that, in the absence of the HPV E6 oncoproteins, E6-AP does not play a prominent role in p53 degradation, it seems likely that the observed increase in p53 levels is an indirect rather than a direct effect of loss of E6-AP expression.

EDD mRNA is frequently overexpressed in breast and ovarian cancers [81,82]. In addition, the *EDD* gene was reported to be mutated in mammary ductal carcinoma [83]. Since the *hyd* gene, which encodes the proposed *Drosophila* ortholog of EDD, was originally identified as a tumor suppressor gene [131], these findings indicate that deregulation of EDD activity could contribute to tumor development. However, etiological association of EDD with human disease remains to be shown. Conventional EDD null mice (generated by the Watts Group, Garvan Institute of Medical Research, Australia) are embryonic lethal [132], though the molecular mechanisms leading to embryonic death remain unclear. Thus, the generation of conditional transgenic mouse models and further characterization of the molecular functions of HYD in *Drosophila*[131,133] will be important in the elucidation of the role of EDD in cell regulatory pathways.

As discussed in *Localization and Function - SI-HECT E3s*, HECTH9 is overexpressed in various cancers and acts as a positive effector of cell proliferation, suggesting that the protein could be a promising target for anti-cancer therapies. This notion is supported by the observations that HECTH9 (i) plays an important role in p53 degradation; (ii) is negatively regulated by the human tumor suppressor p14ARF and (iii) activates the proto-oncoprotein c-Myc. Furthermore, RNAi-mediated downregulation of HECTH9 expression interferes with the growth of p53 null cells (summarized in [95]). Taken together, these observations indicate that HECTH9 has both p53-dependent and p53-independent pro-proliferative properties. Whether or not the p53-independent properties of HECTH9 are related to its activation of c-Myc and whether p14ARF affects c-Myc activation remain to be determined. Furthermore, it should be noted that the observation that HECTH9 targets the anti-apoptotic protein Mcl-1 for degradation [94] is not consistent with its pro-proliferative properties. In any case, the generation of conventional and conditional transgenic mouse models for the murine ortholog of HECTH9 will provide valuable insight into the role of this protein in cell regulatory pathways.

## Disease targets and ligands

As discussed in this review, several HECT E3s have been proposed to be involved in the development of human disease. However, ligands targeting HECT E3s have not yet been described, which may (at least in part) be due to the fact that there is only limited information to date on both physiologically relevant substrate proteins of HECT E3s and the actual physiological function of HECT E3s (i.e. which HECT E3s are involved in which cellular pathways). Thus, in the following, we briefly discuss those HECT E3s that, based on current knowledge, may represent potential targets for therapeutic approaches.

### HERC E3s

The HERC1 target protein TSC2 [21] has been associated with the development of the hereditary disorder tuberous sclerosis complex (TSC). Furthermore, the half-life of TSC2 appears to be significantly shortened in TSC patients either by mutation in the *TSC2* gene or by loss of expression of TSC1, which binds to and stabilizes TSC2 [21]. Thus, if TSC2 stability is mainly controlled by HERC1 (which remains to be shown), HERC1 represents a putative target in the treatment of TSC.

Although HERC5 has not yet been associated with human disease, the available data indicate that HERC5 plays an important role in the immune response [27,28] and could thus represent a potential therapeutic target. However, the actual physiological function and physiologically relevant substrate proteins of HERC5 have not yet been delineated.

### Nedd4/Nedd4-like E3s

There is considerable evidence supporting the notion that the activity of ENaC is regulated by Nedd4-2 and deregulated in many cases of Liddle's syndrome [30,45,97-100,102]. However, since deregulation of ENaC activity in Liddle's syndrome patients is due to the inability of the respective ENaC mutants to interact with Nedd4-2, ENaC rather than Nedd4-2 appears to be a relevant target for therapeutic approaches.

Smurf2 has been associated with the development of tumors. However, the notion that both inappropriate activation and inactivation of Smurf2 could contribute to cancerogenesis [33,111,112] will need to be taken into account when considering Smurf2 as a potential target for therapeutic approaches.

Genetic experiments in mice indicate that Smurf1 negatively controls osteoblast activity and that Smurf1 inactivation does not affect skeletal integrity. Thus, if Smurf1 plays a similar role in humans, then inactivation of the protein could prove beneficial for the treatment of age-related bone loss.

### SI-HECT E3s

E6-AP has been etiologically associated with the development of AS [69-72,125,126]. However, since loss of E6-AP activity is observed in AS patients, substrate proteins of E6-AP rather than E6-AP itself represent targets for therapeutic approaches. In addition, E6-AP is hijacked by the E6 oncoprotein of cancer-associated HPVs to deregulate the activity of several important cell regulatory proteins [74-78]. Thus, the E6/E6-AP interaction could provide a feasible target for molecular approaches in the treatment of cervical cancer. However, in this particular case, E6 rather than E6-AP appears to be the target of choice, for obvious reasons [78].

Similar to E6-AP, deregulation of EDD [81-83] and HECTH9 (summarized in [95]) has been associated with cancerogenesis. Thus, interfering with the interaction between these proteins and their respective substrate proteins (e.g. EDD and Paip2, HECTH9 and p53), or with their E3 activity, could prove beneficial in the treatment of the respective cancers.

## New frontiers in drug discovery

Although HECT E3s are (potentially) involved in the development of human disease, it remains to be determined if HECT E3s can indeed serve as targets for molecular therapeutic approaches. To do so, the following issues need to be addressed:

(i) What are the substrates of the respective E3? In which pathways is the respective E3 involved and in which tissues? This is certainly one of the most challenging tasks and requires extensive proteomic efforts. In fact, the physiologically relevant substrate proteins of most HECT E3s are not yet known and their identification may be hampered by the notion that a given protein is recognized as a substrate by more than one E3. However, knowledge of the substrates and the networks involving the respective E3s will be invaluable when designing molecular therapies.

(ii) Is inactivation or inappropriate activation of HECT E3 activity involved in disease development? If the former is the case, potential therapeutic strategies will further depend on whether E3 activity is lost by genetic mutation (e.g. loss of E6-AP activity in AS) or by alteration of the activity of regulators of the respective E3. In such cases, the substrate proteins or proteins that regulate the activity of HECT E3s (e.g. Sgk1 kinase, which negatively affects Nedd4-2/substrate interaction) rather than the E3s themselves may be the most appropriate targets. If inappropriate activation of a HECT E3 contributes to disease, the HECT E3 itself (e.g. overexpressed HECTH9 in cancer) or activators of the E3 activity (e.g. the E6/E6-AP interaction in cervical cancer) could be the targets of choice.

(iii) How to target HECT E3s? In principal, interference with E3 activity can be envisioned at two levels; by targeting the substrate/E3 interaction or by targeting the catalytic activity of HECT E3s (i.e. the HECT domain). The notion that the former strategy can be successfully used is supported by approaches aimed at interfering with the interaction between p53 and one of its main regulators, the RING finger E3 ligase Mdm2. In this case, it has been shown that small molecules that inhibit the interaction of p53 with Mdm2 induce p53 accumulation and activation in tumor cells, and thus interfere with the growth of wild-type p53-expressing tumor cells [134]. Similar strategies can be envisioned for HECTH9, which appears to be overexpressed in various tumors. Due to the notion that HECTH9 has both p53-dependent and p53-independent pro-proliferative activities, interference with HECTH9 activity could provide a strategy that may be applicable in the treatment of tumors independent of their p53 status.

At first glance, strategies targeting the HECT domain appear to be less attractive, since such molecules may be rather unspecific insofar as they could interfere with the activity of HECT E3s in general. However, structural studies have shown that, when compared with the E6-AP HECT domain, the HECT domain of Smurf2 has a suboptimal E2 binding pocket and that Smad-7 stimulates the E3 activity of Smurf2 by recruiting UbcH7 to the HECT domain [4,6]. Thus, it may be possible to identify and develop small molecules that are specific inhibitors of distinct HECT domains.

Regardless of whether the HECT domain or the E3/substrate interaction may be the target of choice, extensive structural studies are clearly required to obtain insight into the molecular details of the respective E3/substrate interactions and of HECT E3-catalyzed ubiquitylation of substrate proteins. Such information will be invaluable when designing ligands targeting the respective HECT E3.

## Abbreviations

PWS, Prader-Willi syndrome; AS, Angelman syndrome; TCR, T-cell receptor; HPV, human papillomavirus.

## Competing interests

The authors declare that they have no competing interests.

## Publication history

Republished from Current BioData's Targeted Proteins database (TPdb; ).
